# Supplementary material for: Facing obesity in pain rehabilitation clinics: Profiles of physical activity in patients with chronic pain and obesity—A study from the Swedish Quality Registry for Pain Rehabilitation (SQRP)
Source: PLoS One. 2020 Sep 28;15(9):e0239818. doi: 10.1371/journal.pone.0239818 (PMC7521725; doi:10.1371/journal.pone.0239818)
Supplement: S1 Appendix — (DOCX) [file pone.0239818.s001.docx]

# S1 Appendix. Orthogonal Partial Least Square Regressions (OPLS) statistical method

In order to confirm the regressions presented in tables 2-3 Orthogonal Partial Least Square Regressions (OPLS) were done. These are based on other assumptions than the regressions applied in the manuscript. OPLS analysis takes advantage of the fact that variables may be intercorrelated and can up to a certain level handle missing data. On the other hand, it is not possible to determine the effect of a certain variable when controlling for other variables. However, the important independent variables will be possible to identify.

Classical regression techniques assume variable independence when interpreting results [1, 2]. If multicollinearity occurs among the X-variables, the regression coefficients may become unstable and their interpretability breaks down. SIMCA-P+ (version 15 (Umetrics, Sartorius Stedim Biotech, Umeå) uses the Nonlinear Iterative Partial Least Squares algorithm (NIPALS algorithm) when compensating for missing data: max 50% missing data for variables/scales and max 50% missing data for subjects. In the context of the obvious risks for multicollinearity problems (e.g. intercorrelations between the psychological variables investigated). In these confirmatory analyses we used advanced Principal Component Analysis (PCA) for the multivariate correlation analyses to detect outliers and Orthogonal Partial Least Square Regressions (OPLS) for the multivariate regressions using SIMCA-P+. These methods do not require normal distribution [3]. Variables were unit variance (UV) scaled prior to the analyses.
PCA was used to check for multivariate outliers; this was done since outliers can markedly bias regressions. R^2^ describes the goodness of fit – the fraction of sum of squares of all the variables explained by a principal component. Q^2^ describes the goodness of prediction – the fraction of the total variation of the variables that can be predicted by a principal component using cross validation methods [2]. Outliers were identified using two methods: 1) score plots in combination with Hotelling’s T^2^, and 2) distance to model in X-space [2].
No extreme outliers were detected in the present study.

OPLS was used to explore the relative roles of sociodemographic characteristics including BMI, pain aspects and psychological factors for the PA time in quartiles [2]. When regressing the dichotomous insufficient PA-time OPLS discriminant analysis (OPLS-DA) was applied. The variable influence on projection (VIP) indicates the relative relevance of each X-variable. VIP ≥ 1.0 was considered significant if the VIP value had a 95% jack-knife uncertainty confidence interval non-equal to zero [2]. P(corr) was used to note the direction of the relationship (positive or negative). P(corr) depicts the loading of each variable scaled as a correlation coefficient, thus standardizing the range from -1 to +1. P(corr) is stable during iterative variable selection and comparable between models. An absolute P(corr) > 0.4-0.5 is generally considered significant [3]. For each regression, we report the R^2^, Q^2^, and the result (i.e., p-value) of a cross-validated analysis of variance (CV-ANOVA). In the present study we required significant CV-ANOVA for a regression to be significant. A certain variable was considered a significant variable when VIP > 1.0 and absolute p(corr) > 0.40.

# References:

1. Pohjanen E, Thysell E, Jonsson P, Eklund C, Silfver A, Carlsson IB, et al. A multivariate screening strategy for investigating metabolic effects of strenuous physical exercise in human serum. J Proteome Res. 2007;6(6):2113-20. Epub 2007/04/13. doi: 10.1021/pr070007g. PubMed PMID: 17428078.

2. Eriksson L, Byrne T, Johansson E, Trygg J, Vikström C. Multi- and Megavariate Data Analysis: Basic Principles and Applications. Third revised edition ed. Malmö: MKS Umetrics AB; 2013.

3. Wheelock AM, Wheelock CE. Trials and tribulations of 'omics data analysis: assessing quality of SIMCA-based multivariate models using examples from pulmonary medicine. Mol Biosyst. 2013;9(11):2589-96. doi: 10.1039/c3mb70194h. PubMed PMID: 23999822.
