# Supplementary material for: Facing obesity in pain rehabilitation clinics: Profiles of physical activity in patients with chronic pain and obesity—A study from the Swedish Quality Registry for Pain Rehabilitation (SQRP)
Source: PLoS One. 2020 Sep 28;15(9):e0239818. doi: 10.1371/journal.pone.0239818 (PMC7521725; doi:10.1371/journal.pone.0239818)
Supplement: S1 Table — (DOCX) [file pone.0239818.s002.docx]

# S1 Table. Characteristics of patients included in logistic regression (N=2299) and excluded cases (missing BMI n= 505, and dropouts n=811).

|  | Cases included in logistic regressions, N=2299 | Excluded cases n=1316 ^1^ | P value |
| --- | --- | --- | --- |
| BMI, Mean±SD | 27.2±5.4 | 26.8±5.2 | 0.163 |
| BMI category |  |  | 0.112 |
| Underweight | 29 (1.3) | 17 (2.1) | - |
| Normal weight | 860 (37.4) | 306 (37.7) | - |
| Overweight | 814 (35.4) | 299 (36.9) | - |
| Obesity I | 403 (17.5) | 140 (17.3) | - |
| Obesity II-III | 193 (8.4) | 49 (6.0) | - |
| Women, n (%) | 1717 (74.7) | 1015 (77.1) | 0.1 |
| Age, years, Mean±SD | 44±12.3 | 46.0±11.9 | **<0.001** |
| Born outside Europe, n (%) | 263 (11.4) | 189 (14.4) | **0.011** |
| University/college, n (%) | 630 (27.4) | 316 (24) | **0.026** |
| Work/study, n (%) | 1230 (53.5) | 515 (41.4) | **<0.001** |
| NRS-7days, Mean±SD | 6.9±1.7 | 6.7±2.2 | 0.526 |
| PRI, Mean±SD | 14.8±8.5 | 15.2±9.1 | 0.420 |
| Pain-duration, months, median (q1-q3) | 156 (65-396) | 188 (69-416) | 0.038 |
| Presence of contant pain, n (%) | 1906 (82.9) | 907 (68.9) | **<0.001** |
| HADS-A, Mean±SD | 9.4±4.7 | 9.5±4.8 | 0.408 |
| HADS-D, Mean±SD | 9.1±4.5 | 9.3±4.5 | 0.133 |
| Insomnia index, Mean±SD | 16.7±6.7 | 16.9±6.5 | 0.402 |
| RAND-36 MCS, Mean±SD | 41.4±21.5 | 38.9±20.9 | 0.019 |
| MPI-affective distress, Mean±SD | 3.5±1.3 | 3.5±1.3 | 0.891 |
| MPI-lifecon, Mean±SD | 2.7±1.1 | 2.7±1.2 | 0.473 |
| PA time /week, minutes, Median (q1-q3) | 165 (75-300) | 135 (45-300) | **0.022** |
| Insufficient PA, n (%) | 1069 (46.5) | 493 (50.4) | **0.04** |

^1^ =missing data in each variable excluded in the analysis
