# Supplementary figures and images for: Facing obesity in pain rehabilitation clinics: Profiles of physical activity in patients with chronic pain and obesity—A study from the Swedish Quality Registry for Pain Rehabilitation (SQRP)
Source: PLoS One. 2020 Sep 28;15(9):e0239818. doi: 10.1371/journal.pone.0239818 (PMC7521725; doi:10.1371/journal.pone.0239818)

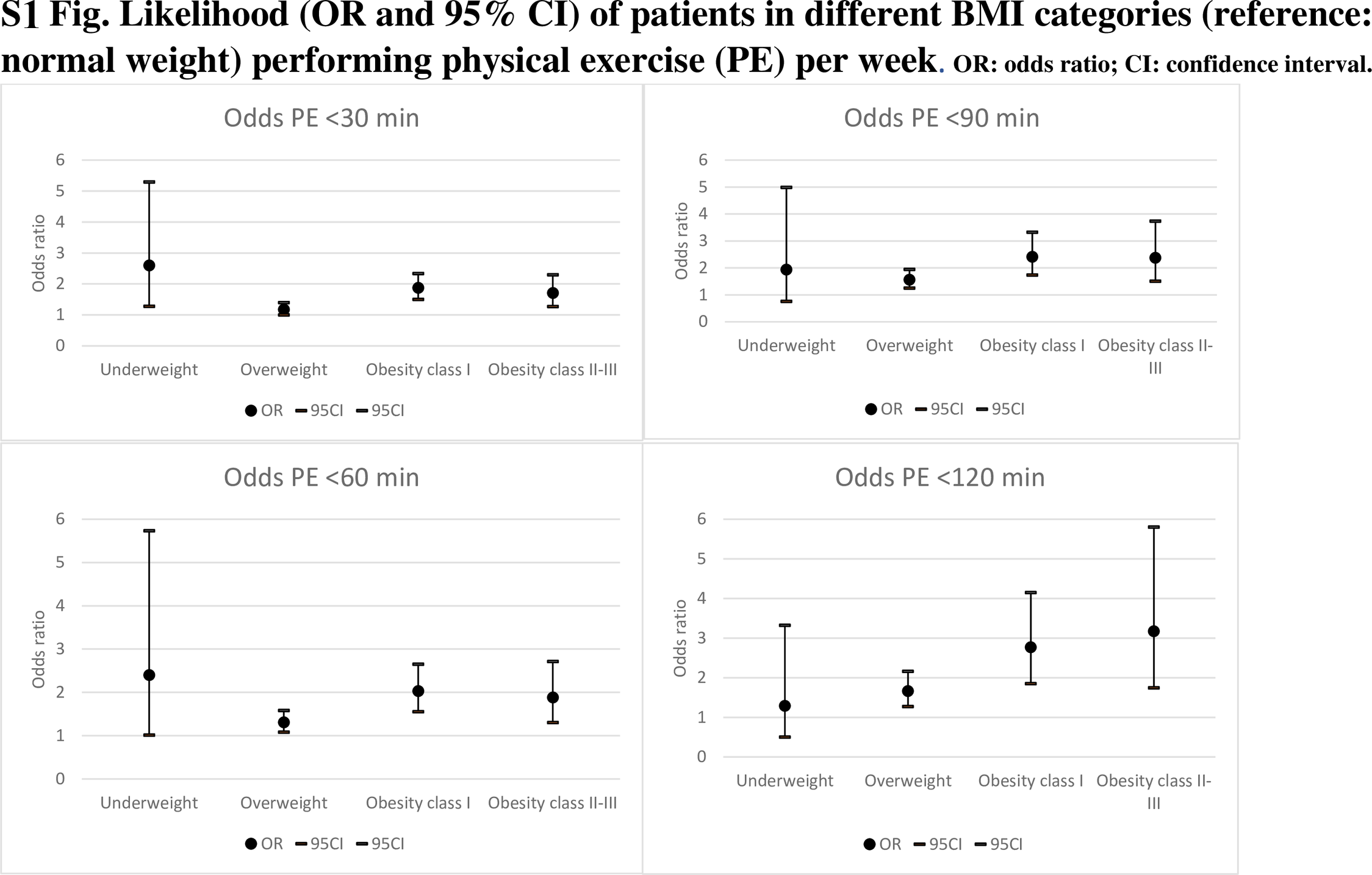

Supplement: S1 Fig — (TIF) [file pone.0239818.s003.tif]

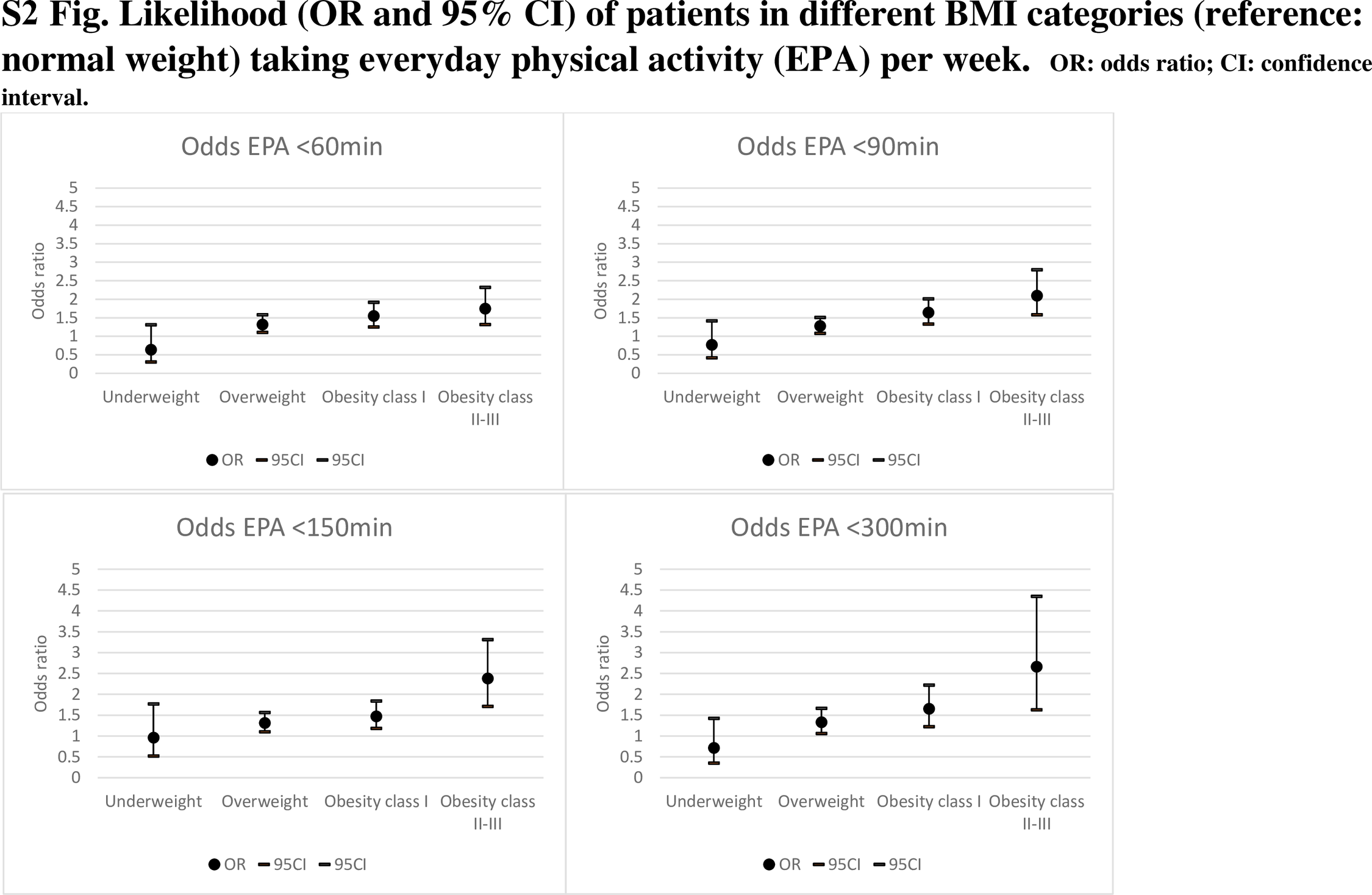

Supplement: S2 Fig — (TIF) [file pone.0239818.s004.tif]
